# Trust in adolescence and depression and anxiety symptoms in young adulthood: findings from a Swedish cohort

**DOI:** 10.1186/s13104-023-06667-7

**Published:** 2024-01-02

**Authors:** Sara Brolin Låftman, Jonas Raninen, Viveca Östberg

**Affiliations:** 1 Department of Public Health Sciences, Centre for Health Equity Studies (CHESS), Stockholm University, SE-106 91 Stockholm, Sweden; 2 Department of Clinical Neuroscience, Karolinska Institutet, SE-171 77 Stockholm, Sweden; 3 Centre for Alcohol Policy Research, La Trobe University, Melbourne, Australia

**Keywords:** Generalised trust, Institutional trust, Depression, Anxiety, Young adulthood

## Abstract

**Objective:**

Trust and health are both fundamental elements of a socially sustainable society. While much research has shown that trust is associated with better mental health outcomes in adults, studies of young people are relatively scarce, despite the fact that mental health problems are common in young ages. In particular, there are few longitudinal studies that cover different dimensions of trust. Building on a previous study on trust and psychosomatic complaints in adolescents, the aim was to examine the links between generalised and institutional trust in adolescence and depression and anxiety symptoms in young adulthood. Data was obtained from a Swedish cohort study with self-reported information on generalised and institutional trust at ages 15–16 and 17–18 and depression and anxiety symptoms at age 20–21 (n = 2,668). Covariates included sociodemographic characteristics and indicators of prior mental health status.

**Results:**

Binary logistic and linear regressions showed that higher levels of generalised trust at ages 15–16 and 17–18 were inversely associated with depression and anxiety symptoms at age 20–21. Institutional trust was however not linked with subsequent depression and anxiety symptoms when adjusting for generalised trust and covariates. The findings indicate that generalised trust is a social determinant for mental health in young people.

## Introduction

Trust and health are both fundamental elements of a socially sustainable society. Two key dimensions of trust are trust in other people (which is often referred to as generalised, horisontal, social, or interpersonal trust), and trust in the public institutions of society (often referred to as institutional or vertical trust). A significant amount of research has demonstrated that trust in other people is associated with better health outcomes in adults [1–6]. Inquiry into the links between trust in public institutions and health is less common, but studies have shown that political trust (as one aspect of institutional trust) is associated with better health outcomes even when adjusting for trust in other people [7–9], even though the direction of the association is unclear [10].

Trust can be expected to be associated with mental health due to several factors. Generalised trust may enhance access to social support [2], which in turn is associated with better health both through its direct effects and as a buffer against stress [11]. By contrast, individuals who have low trust in others may experience heightened levels of stress as they may perceive social interactions as potentially threatening or unreliable [2, 12]. Institutional trust may be linked to a sense of belonging to a community and the belief that the community operates for the collective well-being of everyone in society, potentially contributing to enhanced mental health [7].

In Sweden, mental health problems are prevalent among adolescents and young adults [13], making it pertinent to identify social determinants at various levels. However, studies focusing on the relationship between trust and health in young people are relatively scarce (but see [14–18]). Notably, there are few longitudinal studies that encompass different dimensions of trust.

In a previous article, we showed that generalised and institutional trust were inversely associated with psychosomatic complaints in a Swedish cohort of adolescents [19]. Using more recently collected data from the same cohort, the aim of the current study was to examine the prospective links between generalised and institutional trust in adolescence (age 15–16 and 17–18) and depression and anxiety symptoms in young adulthood (age 20–21), whilst also adjusting for sociodemographic characteristics, including gender, as well as indicators of prior mental health problems.

## Methods

Data was obtained from the Swedish national cohort study Futura01. The sampling procedure was school-based and included 500 schools across Sweden, of which 343 agreed to participate. One class was randomly selected in each school. Wave 1 was conducted in classrooms in 2017 when participants attended grade 9 (age 15–16) (n = 5,537). Wave 2 was carried out in 2019 as a web survey or postal survey, when participants typically attended the second grade of upper secondary school (age 17–18) (n = 4,141). Wave 3 was conducted as a web survey in 2022, after the majority of participants had finished upper secondary school (age 20–21) (n = 3,193). In all, 2,836 individuals participated in all three surveys with a somewhat higher attrition among boys and among adolescents whose parents had a lower education or were born abroad. The analytical sample includes 2,668 participants with non-missing information on the study variables. More information on the cohort is provided elsewhere [20].

### Measures

Depression and anxiety symptoms were measured in wave 3 by the Patient Health Questionnaire-4 (PHQ-4), referring to symptoms over the past two weeks [21]. The instrument includes the Patient Health Questionnaire-2 (PHQ-2) with two items on depression symptoms [22] and the Generalized Anxiety Disorder-2 (GAD-2) with two items on anxiety symptoms [23]. We used PHQ-2 and GAD-2 as dichotomous measures, applying the recommended cutoffs at ≥ 3 [21, 24]. However, we also conducted sensitivity analyses using the continuous PHQ-2 and GAD-2 scales, each ranging from 0 to 6. Additionally, we used the full PHQ-4 scale as a continuous measure, ranging from 0 to 12 [21].

The items used to measure generalised and institutional trust were retrieved from the OECD measurement of social capital project and question databank [25] and adjusted to the target population. The measures of generalised and institutional trust have been used previously [19, 26].

Generalised trust was measured in waves 1 and 2 by the question: “Considering society as a whole, mark the alternative that best agrees with how you feel”, and the items (a) “You can trust most people”; (b) “You can never be too careful when you meet new people”; (c) “Most people are trying to be helpful”; (d) “Most people only care about themselves”; and (e) “Most people are honest”. The response categories were “Totally correct”; “Partly correct”; “Partly incorrect”; and “Totally incorrect”, which were coded 4 − 1 (items b and d were reversely coded). The five items showed acceptable internal consistency (Cronbach’s alpha: wave 1: 0.62; wave 2: 0.67). In accordance with previous studies based on the same data [19, 26], we removed item b as this improved the internal consistency (Cronbach’s alpha wave 1: 0.71; wave 2: 0.73). We calculated the mean value of items a, c, d and e for participants who responded to at least three of these. The range was 1–4, with higher values indicating higher levels of generalised trust.

Institutional trust was measured in waves 1 and 2 by the question: “How much do you normally trust…”, and the items (a) “Government and parliament”; (b) “The justice system (police and courts)”; (c) “Teachers”; (d) “News (TV, radio)”; and (e) “Researchers and experts”. The response categories were “Very much”; “Fairly much”; “Not that much”; and “Not at all”, which were coded 4 − 1. The items showed high internal consistency (Cronbach’s alpha wave 1: 0.74; wave 2: 0.72). For respondents who answered at least three of the five items, the mean value of the items was calculated to indicate institutional trust. The range of the measure was 1–4, with higher values indicating higher levels of institutional trust. The correlation between generalised and institutional trust was moderate (Pearson’s r wave 1: 0.36; wave 2: 0.43).

Sociodemographic characteristics included gender (boy or girl), parental education (highest level among parents) and parental country of birth (both parents born outside Sweden or at least one Swedish born parent) based on registry information retrieved in connection with wave 1, and upper secondary programme (vocational or academic) based on survey information in wave 2.

In an effort to address reverse causality, we incorporated indicators of mental health from waves 1 and 2, including medication for depression and anxiety as well as psychosomatic complaints (a summation index including items measuring the frequency of headache, stomach ache, and difficulties to fall asleep; see [19]), based on survey information.

### Statistical analysis

In the analyses of PHQ-2 and GAD-2, binary logistic regressions were performed, presenting odds ratios (OR) and 95% confidence intervals (CI). In the analyses of the full continuous PHQ-4 scale, we conducted linear regressions, presenting unstandardised b coefficients (b) and 95% confidence intervals (CI). For each outcome, we conducted a series of models examining trust measured in wave 1 and wave 2, respectively. We first performed crude models, including one variable at a time, controlling only for gender. Model 1 included generalised trust and covariates; Model 2 included institutional trust and covariates; and Model 3 included both generalised and institutional trust and covariates. To account for the hierarchical nature of the data with students nested in school classes at baseline, robust standard errors were estimated clustering by class in wave 1. The number of classes was 334. All analyses were performed with Stata, version 17 [27].

## Results

Descriptives of the data are presented in Table 1.

**Table 1:** Descriptives. n = 2,668

**Wave 1 (age 15–16)**	n	%		
Gender				
Boys	1113	41.7		
Girls	1555	58.3		
Parental education				
≤ 2 years secondary or less	376	14.1		
≥ 3 years secondary	499	18.7		
Tertiary	1793	67.2		
Parental country of birth				
At least one in Sweden	2275	85.3		
Two parents outside Sweden	393	14.7		
Medication for depression	49	1.8		
Medication for anxiety	58	2.2		
	Mean	s.d.	Min	Max
Psychosomatic complaints	7.03	2.77	3	15
Generalised trust	2.42	0.50	1	4
Institutional trust	2.84	0.53	1	4
**Wave 2 (age 17–18)**	n	%		
Upper secondary programme				
Vocational	491	18.4		
Academic	2103	78.8		
Other programme/other activity/missing	74	2.8		
Medication for depression	124	4.7		
Medication for anxiety	132	5.0		
	Mean	s.d.	Min	Max
Psychosomatic complaints	7.24	2.72	3	15
Generalised trust	2.44	0.51	1	4
Institutional trust	2.83	0.52	1	4
**Wave 3 (age 20–21)**	n	%		
Depression symptoms (PHQ-2, cutoff ≥ 3)	674	25.3		
Anxiety symptoms (GAD-2, cutoff ≥ 3)	730	27.4		
	Mean	s.d.	Min	Max
Depression and anxiety symptoms (PHQ-4, scale 0–12)	3.62	3.00	0	12

The associations between generalised and institutional trust in wave 1 and depression and anxiety symptoms in wave 3 are presented in Table 2. The crude models show that both generalised and institutional trust were inversely and consistently associated with subsequent depression and anxiety symptoms. Sensitivity analyses, including each item of institutional trust separately (not presented), revealed that in a majority of cases, they showed statistically significant associations with the outcomes. The exceptions were that trust in teachers was not associated with PHQ-2 or GAD-2, and trust in researchers and experts was not associated with any of the three outcomes. When adjusting for covariates in Models 1 and 2, the estimates were attenuated. Several of the estimates turned non-significant, but the associations between general trust and PHQ-2 as well as PHQ-4 remained statistically significant. This was true also when mutually adjusting for both dimensions of trust in Models 3. Further analyses removing the item on trust in researchers and experts from the institutional trust scales did not affect the results (not presented in Table). As a sensitivity analysis, we also performed linear regression analyses using the continuous PHQ-2 and GAD-2 scales (not presented in Table). In the fully adjusted models, the estimates for general trust were statistically significant with both outcomes, whereas the estimates for institutional trust were non-significant.

**Table 2:** Associations between generalised and institutional trust in wave 1 (age 15–16) and depression and anxiety symptoms in wave 3 (age 20–21). Results from binary logistic and linear regression models. n = 2,668

	Depression symptoms (PHQ-2, cutoff ≥ 3), wave 3							
	Crude^a^		Model 1^b^		Model 2^c^		Model 3^d^	
	OR	95% CI	OR	95% CI	OR	95% CI	OR	95% CI
Generalised trust, wave 1	0.65***	0.53, 0.78	0.79*	0.65, 0.96			0.79*	0.64, 0.97
Institutional trust, wave 1	0.76**	0.64, 0.90			0.93	0.77, 1.11	0.99	0.82, 1.20
	Anxiety symptoms (GAD-2, cutoff ≥ 3), wave 3							
	Crude^a^		Model 1^b^		Model 2^c^		Model 3^d^	
	OR	95% CI	OR	95% CI	OR	95% CI	OR	95% CI
Generalised trust, wave 1	0.70***	0.58, 0.85	0.87	0.71, 1.06			0.86	0.69, 1.06
Institutional trust, wave 1	0.80**	0.68, 0.94			0.99	0.83, 1.19	1.04	0.86, 1.26
	Depression and anxiety symptoms (PHQ-4, scale 0–12), wave 3							
	Crude^a^		Model 1^b^		Model 2^c^		Model 3^d^	
	b	95% CI	b	95% CI	b	95% CI	b	95% CI
Generalised trust, wave 1	-0.81***	-1.05, -0.58	-0.42***	-0.65, -0.19			-0.45***	-0.70, -0.20
Institutional trust, wave 1	-0.43***	-0.65, -0.21			-0.04	-0.27, 0.18	0.09	-0.15, 0.33

*** *p* < 0.001 **p < 0.01 *p < 0.05

^a^ Includes one variable at a time, controlling for gender

^b^ Includes generalised trust, gender, parental education, parental country of birth, medication for depression and anxiety, and psychosomatic complaints (all measured in wave 1)

^c^ Includes institutional trust, gender, parental education, parental country of birth, medication for depression and anxiety, and psychosomatic complaints (all measured in wave 1)

^d^ Includes generalised and institutional trust, gender, parental education, parental country of birth, medication for depression and anxiety, and psychosomatic complaints (all measured in wave 1)

The associations between generalised and institutional trust in wave 2 and depression and anxiety symptoms in wave 3 are presented in Table 3. In the crude analyses, both dimensions of trust were inversely associated with later depression and anxiety symptoms. Sensitivity analyses with each item of institutional trust separately (not presented) showed that all had statistically significant associations with the outcomes, except for trust in researchers and experts which was not associated with GAD-2 or PHQ-4. Both dimensions of trust were inversely associated with later depression and anxiety symptoms even when adjusting for covariates in Models 1 and 2. However, in Models 3, including both dimensions of trust, the estimates for generalised trust remained statistically significant whereas the those for institutional trust turned non-significant. Again, we performed additional analyses removing the item on trust in researchers and experts from the institutional trust scales, but this did not alter the patterns (not presented in Table). Finally, as an additional check we performed linear regression analyses using the continuous PHQ-2 and GAD-2 scales (not presented in Table). Similar to the analyses of the dichotomous measures, general trust showed statistically significant associations with the continuous PHQ-2 and GAD-2 measures, whereas institutional trust did not.

**Table 3:** Associations between generalised and institutional trust in wave 2 (age 17–18) and depression and anxiety symptoms in wave 3 (age 20–21). Results from binary logistic and linear regression models. n = 2,668

	Depression symptoms (PHQ-2, cutoff ≥ 3), wave 3							
	Crude^a^		Model 1^b^		Model 2^c^		Model 3^d^	
	OR	95% CI	OR	95% CI	OR	95% CI	OR	95% CI
Generalised trust, wave 2	0.49***	0.41, 0.58	0.64***	0.53, 0.77			0.66***	0.54, 0.80
Institutional trust, wave 2	0.60***	0.50, 0.71			0.79*	0.65, 0.96	0.92	0.75, 1.13
	Anxiety symptoms (GAD-2, cutoff ≥ 3), wave 3							
	Crude^a^		Model 1^b^		Model 2^c^		Model 3^d^	
	OR	95% CI	OR	95% CI	OR	95% CI	OR	95% CI
Generalised trust, wave 2	0.57***	0.48, 0.67	0.72***	0.60, 0.87			0.74**	0.61, 0.91
Institutional trust, wave 2	0.66***	0.56, 0.79			0.83*	0.69, 1.00	0.93	0.76, 1.13
	Depression and anxiety symptoms (PHQ-4, scale 0–12), wave 3							
	Crude^a^		Model 1^b^		Model 2^c^		Model 3^d^	
	b	95% CI	b	95% CI	b	95% CI	b	95% CI
Generalised trust, wave 2	-1.19***	-1.40, -0.97	-0.73***	-0.94, -0.51			-0.71***	-0.95, -0.48
Institutional trust, wave 2	-0.77***	-1.00, -0.53			-0.30*	-0.54, -0.06	-0.04	-0.30, 0.22

*** *p* < 0.001 **p < 0.01 *p < 0.05

^a^ Includes one variable at a time, controlling for gender

^b^ Includes generalised trust (measured in wave 2); gender, parental education, parental country of birth (measured in wave 1); and upper secondary programme, medication for depression and anxiety, and psychosomatic complaints (measured in wave 2)

^c^ Includes institutional trust (measured in wave 2); gender, parental education, parental country of birth (measured in wave 1); and upper secondary programme, medication for depression and anxiety, and psychosomatic complaints (measured in wave 2)

^d^ Includes generalised and institutional trust (measured in wave 2); gender, parental education, parental country of birth (measured in wave 1); and upper secondary programme, medication for depression and anxiety, and psychosomatic complaints (measured in wave 2)

## Discussion

Building on a previous article on trust and psychosomatic complaints in adolescents [19], the aim of the present study was to examine the links between generalised and institutional trust in adolescence and depression and anxiety symptoms in young adulthood in a Swedish cohort. The results showed that generalised trust at ages 15–16 and 17–18 was inversely associated with depression and anxiety symptoms at age 20–21. Institutional trust was however not linked with subsequent depression or anxiety symptoms when adjusting for generalised trust and covariates. These results differ somewhat from those reported in our study on trust and psychosomatic complaints based on the same cohort at ages 15–18, which showed that both dimensions of trust had independent associations with psychosomatic complaints [19]. This discrepancy may arise, e.g., from the somewhat different types of outcomes. While psychosomatic complaints are highly prevalent in adolescents in Sweden [28] and may encompass both everyday experiences and health problems [29], our measures of depression and anxiety symptoms specifically serve as ‘yellow flags’ signaling the relevance of a more comprehensive detailed clinical interview for depression and anxiety disorders [24]. Another possibility is that the differing time span between exposures and outcomes may have contributed to different results.

The results of the present study are in line with prior research showing that generalised trust is associated with better health outcomes in adults [1–6] and in young people [14, 16]. Furthermore, the lack of an association between institutional trust and subsequent mental health reflects the results of a cross-sectional American study of disadvantaged youth which did not find any statistically significant association between community or institutional trust and self-rated health [15]. However, our finding is in contrast with cross-sectional analyses of Swedish regional data which showed that political trust (as one aspect of institutional trust) was associated with better health outcomes in adults [7–9].

One interpretation of our findings is that higher levels of generalised trust contribute to greater social support and collective action, thereby assisting individuals in effectively coping with stressors [2, 6], which may in turn reduce the risk of developing mental health problems. Furthermore, low levels of generalised trust can in themselves be stressful [2, 12]. In a study involving upper secondary students in Stockholm [12], the participants described a connection between distrust towards others and feelings of stress and exhaustion, and one student argued that “you would probably get paranoid if you go around thinking that nobody can be trusted” (p. 214). While low levels of institutional trust may also be perceived as stressful [7], the lack of statistically significant relationships between institutional trust and subsequent depression and anxiety symptoms in the fully adjusted models suggests that generalised trust plays a relatively more important role in these outcomes. One possibility is that generalised trust is associated with social support [2], a fundamental resource in people’s everyday lives and a key determinant of mental health [30–32], and therefore has a greater impact than institutional trust. This would imply that generalised trust is more proximal to the outcomes, compared to institutional trust, and therefore remains of significant importance in a model including both measures. It should however be noted that both measures of trust were significantly associated with all three outcomes in the crude models

## Conclusion

The findings of the present study indicate that generalised trust is a social determinant for mental health in young people. Considering this result, as well as the importance of trust in creating a well-functioning and socially sustainable society, it is relevant to ask how trust can be induced and strengthened in this age group. One important arena is the school. For instance, previous research has recognised that a positive school climate characterised by features such as openness, fairness, compassion, lack of conflicts, and lack of bullying can strengthen young persons’ trust in other people [33, 34].

## Limitations

In the baseline (wave 1) data collection there was non-response at the school level, although it should be highlighted that there were no statistically significant differences between participating and non-participating schools with regards to average school performance, the proportion of university educated parents, or the proportion of parents born abroad [20]. Furthermore, there was attrition between waves of data collection, with 58% of the full wave 1 sample participating in wave 3. Importantly, comparing descriptives of the full wave 1 and wave 2 samples with those of our analytical sample (including data from all three waves) showed there was a certain degree of bias in that females, those whose parents had a university education and those whose parents were not born abroad were more likely to participate in all three surveys. This bias may have affected the generalisability of our findings.

Another limitation concerns the fact that we have information on depression and anxiety symptoms only in wave 3, and hence we were not able to control for the prevalence of these symptoms in waves 1 and 2 when trust was measured. In an attempt to take earlier mental health problems into account, we therefore adjusted for self-reported psychosomatic complaints, which are known to be associated with both trust [19] and depression and anxiety symptoms [35], as well as medication for depression and anxiety. Nonetheless, it is still possible that prior depression or anxiety symptoms may have affected the associations, and/or that there are reciprocal links between trust and depression and anxiety symptoms (cf. [19]).

Finally, it should be acknowledged that our measure of institutional trust did not include any item on trust in the healthcare system, which could be considered a limitation. Additionally, our dataset lacked measures of urbanity, posing another potential limitation as it may be a confounding factor.

The findings of the study should be considered with the above-mentioned limitations in mind.

## Data Availability

The data used for the current study are available from Karolinska Institutet but restrictions apply to the availability of these data, and are therefore not publicly available. Data are however available from the Principal Investigator Dr. Jonas Raninen (jonas.raninen@ki.se) upon reasonable request and with permission of Karolinska Institutet and ethical approval from the Swedish Ethical Review Authority.

## Abbreviations

CI: Confidence interval
GAD-2: Generalized Anxiety Disorder-2
OECD: Organisation for Economic Co-operation and Development
OR: Odds ratio
PHQ-2: Patient Health Questionnaire-2
PHQ-4: Patient Health Questionnaire-4
